# A systematic exploration of current limitations of cognate-based phylogenetic inference

**DOI:** 10.12688/openreseurope.20351.3

**Published:** 2026-01-28

**Authors:** Luise Häuser, Gerhard Jäger, Alexandros Stamatakis

**Affiliations:** 1 Karlsruhe Institute of Technology Institute of Theoretical Informatics, Karlsruhe, Baden-Württemberg, Germany; 2 Computational Molecular Evolution, Heidelberg Institute for Theoretical Studies, Heidelberg, Baden-Württemberg, Germany; 3 Seminar für Sprachwissenschaft, University of Tübingen, Faculty of Humanities, Tübingen, Baden-Württemberg, Germany; 4 Biodiversity Computing Group, Foundation for Research and Technology - Hellas, Institute of Computer Science, Heraklion, Crete, Greece

**Keywords:** Phylogenetic Inference, Historical Linguistics, Maximum Likelihood, Evolutionary Model, Cognate Data, Machine Learning, Phylogenetic Difficulty

## Abstract

**Background:**

Computational tools for phylogenetic inference are now routinely applied to data from historical linguistics, especially cognate data.

**Methods:**

We initially provide an overview of the cognate datasets that are publicly available at present and compare the amount of cognate data with the available masses of molecular data. Then, we outline the drawbacks of the standard binary cognate data representation and introduce an alternative representation that alleviates some of these disadvantages. We also introduce dedicated, parameter-rich evolutionary models for this novel representation. We implement the model and investigate its behavior. In addition, we conduct an orthogonal experiment to investigate whether machine learning-based approaches can be used for cognate data.

**Results:**

Our experiments show that our newly introduced models can currently not be applied, as they exhibit clear indications for overparameterization due to the small size of the available cognate datasets. We demonstrate that, for the same reason, the applicability of emerging machine learning-based approaches to cognate data is highly limited.

**Conclusion:**

We conclude that it is necessary to collect more data, investigate potential data sources, and also consider alternative types of data. Historical linguistics will be able to benefit from recent advances in phylogenetics if the amount of available datasets can be substantially increased, both, in terms of number of datasets, and dataset sizes.

## Plain Language Summary

Scientists working on phylogenetics aim to construct phylogenetic trees that represent the evolutionary relationships between different species. Numerous computational tools exist for reconstructing these relationships. Some of these tools rely on sophisticated mathematical models or machine learning-based approaches. Such tree reconstruction methods can be applied because large amounts of molecular data are available due to advances in biological wet-lab sequencing techniques. Phylogenetic trees also play an important role in historical linguistics, where they represent the evolutionary relationships between languages rather than between species. It is comparatively straight-forward to apply the tree reconstruction methods used for biological data to linguistic data. However, the datasets that are being analyzed in historical linguistics need to be manually assembled (and not automatically by a DNA sequencing device) and are therefore smaller in size and numbers. We introduce a new approach for encoding language data for phylogenetic inference and for modeling the process of language evolution. By means of computational experiments we show however, that the currently available linguistic datasets are too small to apply this novel approach. In another experiment we demonstrate that machine learning approaches can also not be transferred from molecular phylogenetics to historical linguistics without restrictions. Our work shows that the field of historical linguistics needs to generate substantially more data in order to use more involved approaches for reconstructing the evolutionary history of languages.

## 1 Introduction

Computational phylogenetic methods are by now routinely deployed in the field of historical linguistics. While phylogenetic methods can be applied to different types of linguistic input data ( Dediu & Cysouw, 2013), cognate data are most widely used. Each cognate dataset relies on a concept list, such as the Swadesh-100-List ( Swadesh, 1955), for instance. When assembling data for a specific language, linguists attempt to identify a commonly used every-day word for each concept that describes the specific concept as accurately as possible ( Dunn, 2013). This data collection process substantially differs from the largely automated data generation process in molecular biology. Hence, linguistic datasets are substantially smaller in size and number (see Section 2).

Phylogenetic inferences on cognate data have predominantly been conducted via Bayesian Inference (BI) methods ( Heggarty *et al.*, 2023; Kolipakam *et al*., 2018; Sagart *et al*., 2019), but Maximum Likelihood (ML) based tree inferences have been used as well ( Jäger, 2018), in particular for reconstructing extremely large language trees. Note that, ML and BI both rely on exactly the same type of phylogenetic likelihood calculations. Cognate data are typically encoded as binary character matrices ( Häuser *et al.*, 2024a) which are subsequently provided as input to phylogenetic inference tools.

Despite its simplicity, this binary representation also exhibits some drawbacks (see Section 3.1). This raises the question if cognate data can be encoded in a more sophisticated manner, which will in turn also require a distinct evolutionary model. We introduce such a representation along with appropriate evolutionary models. These models however comprise more free parameters that in turn, as we show here, will require a larger amount of cognate data to be reliably estimated in order to circumvent overparametrization.

In addition, numerous recent methodological advances in molecular phylogenetics rely on machine learning methods ( Azouri *et al*., 2021; Haag *et al*., 2022; Nesterenko *et al*., 2024; Trost *et al*., 2024). We show that larger datasets will also be required to apply these advances to language data.

We conclude that recent advances in molecular phylogenetics, such as more sophisticated and parameter-rich models as well as machine learning-based approaches, can currently not be applied to cognate data, and we also advise against doing so. To move forward, we therefore recommend to focus on acquiring more data before further improving language tree inference methodology.

The remainder of this paper is organized as follows: In Section 2 we first provide an overview of the currently available amount of cognate data and compare it to the available amount of molecular data that is several orders of magnitude larger. We also analyze how the respective linguistic and molecular datasets differ with respect to their information content. Then, we introduce our novel representation for cognate data and the corresponding evolutionary models. We analyze the performance of these models on existing cognate data and demonstrate why more data is required to deploy these more complex (parameter-rich) models in linguistics ( Section 3). Using the example of Pythia ( Haag *et al*., 2022), a machine learning-based tool for molecular phylogenetics, we conduct experiments illustrating that the amount of cognate data currently available is insufficient for training even comparatively simple machine learning based approaches ( Section 4).

## 2 Available data

**Figure 1.**
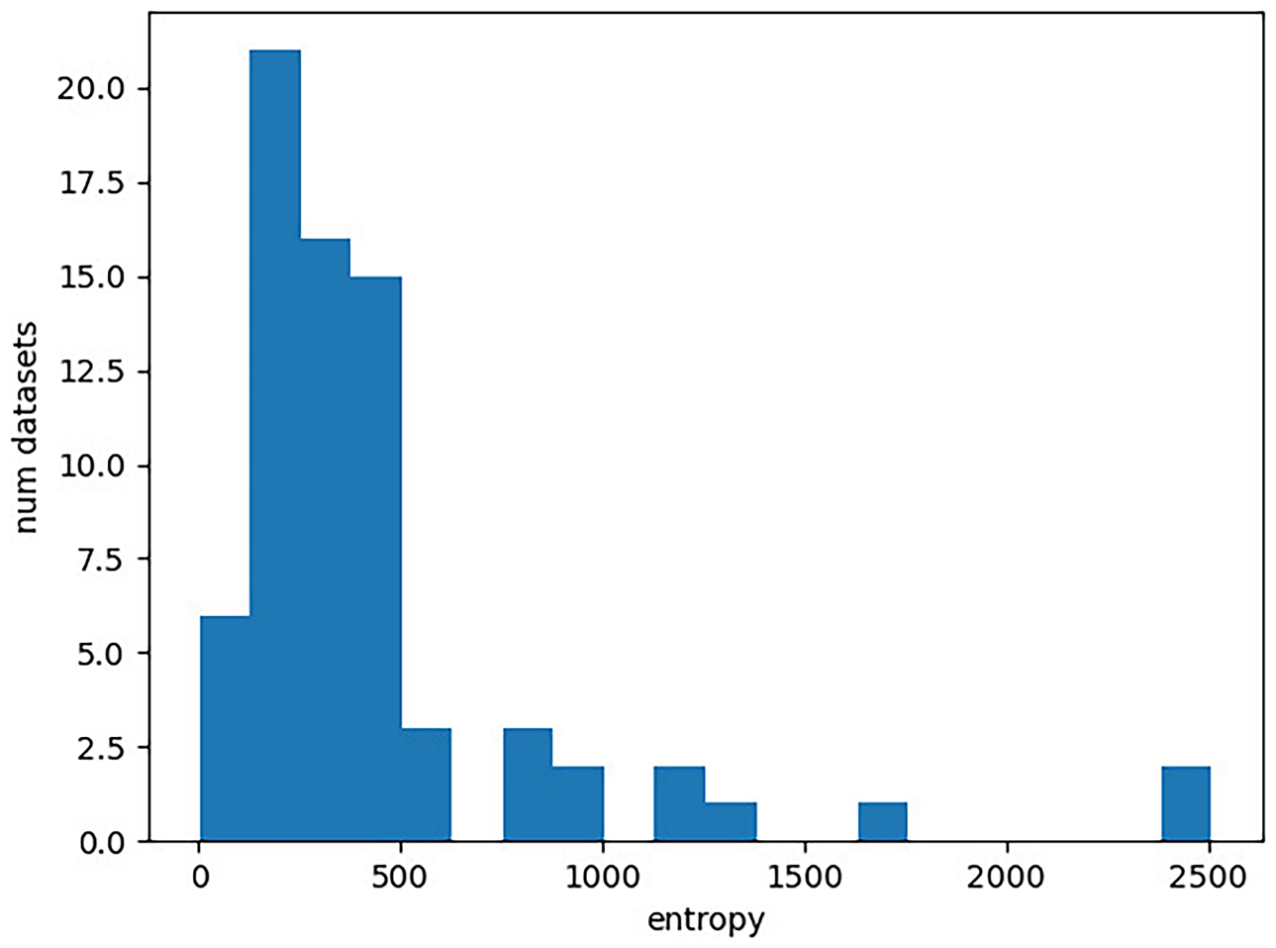
Distribution of entropies for the Lexibench datasets. The x-axis gives the entropy of a character matrix, which we obtain as the sum over the per-column Shannon entropies. The y-axis corresponds to the number of Lexibench datasets with the respective entropy value.

To illustrate the aforementioned cognate data sparsity, we analyze the available amount of manually assembled cognate data and compare it to the amount of available molecular data. To this end, We use the cognate datasets published in *Lexibench* ( Häuser & List, 2025) which comprises a collection of appropriate benchmark data. We consider these datasets to exhibit high data quality and therefore be suitable for phylogenetic inference. In DNA data, each column corresponds to a specific character position in the genome, whereas in cognate data, a group of binary columns represents a concept ( Häuser *et al.*, 2024a). Therefore, for comparing the amount of information in the datasets, it does not suffice to count the number of columns in the character matrices. Instead, we calculate the per column Shannon entropy ( Shannon, 1948) and sum it over all columns in the character matrix. The distribution of these entropies for the Lexibench datasets is illustrated in Figure 1. In the plot, the x-axis corresponds to the entropy of a character matrix calculated as described above. The y-axis gives the number of Lexibench datasets with the respective entropy value.

**Figure 2.**
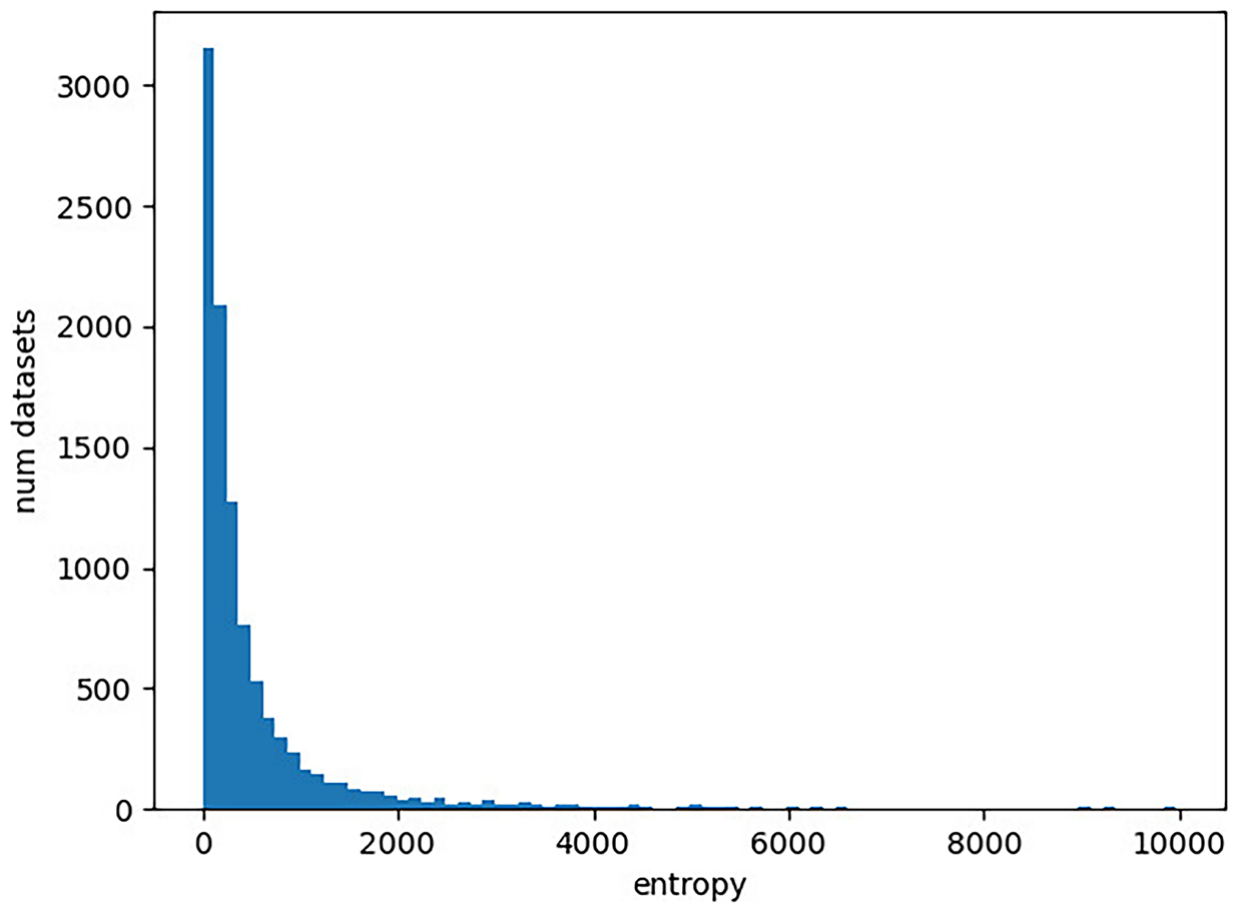
Distribution of entropies for DNA character matrices in TreeBase. The x-axis gives the entropy of a character matrix, which we obtain as the sum over the per-column Shannon entropies. The y-axis corresponds to the number character matrices in TreeBase with the respective entropy value. Note that there are 193 character matrices with an entropy exceeding 10,000, which are not included in the plot for improved visualization.

In the following, we conduct a comparison with molecular data. For this purpose, we use a set of 10,406 DNA character matrices that were published as part of research papers and submitted to the *TreeBase* ( Piel *et al*., 2009) repository by evolutionary biologists. Note that TreeBase only contains a minuscule fraction of the overall amount of molecular data available. The *EvoNaps* ( Reden, 2023) database created for machine learning and data exploration purposes comprises more than 29,000 biological character matrices. Additionally, new DNA character matrices can seamlessly be automatically assembled using data provided by the National Center for Biotechnology Information (NCBI, https://www.ncbi.nlm.nih.gov/). In our experiment, we determine the entropy of each character matrix in TreeBase as described above. We provide the entropy distribution in Figure 2. The corresponding Python script is available online ( Häuser, 2025a [Code]).

First, we observe that there is a substantially larger number of molecular datasets available, even when restricting ourselves to TreeBase. This is particularly important for the application of machine learning methods where large quantities of datasets are required as training data. We currently simply lack the necessary amount of cognate data for any machine learning endeavors.

The entropy ranges of the datasets are similar for both DNA data and cognate data. In particular, the cognate datasets do not yield higher entropy values. This indicates that appropriate models of language evolution should not comprise more free parameter than models of DNA evolution. It is therefore not possible to apply very complex approaches for modeling the evolution of languages’ vocabulary, given the available amount of cognate data.

## 3 Modeling cognate data

To conduct phylogenetic inference on cognate data, they need to be represented in an appropriate manner. Further, a substitution model is required to describe the underlying evolutionary processes for such a data representation. In the following we introduce a novel data representation and corresponding novel substitution models that are tailored to cognate data. We initially motivate this endeavor in section 3.1. Next, we formally describe cognate data and its standard binary representation (see Section 3.2.1). We then go into detail concerning the novel bit-vector based representation Section 3.2.2) and we outline the fundamental assumptions we make to describe the evolutionary process of this data type (see Section 3.2.3). In Section 3.3, we initially explain the standard substitution models used in our experiments. Then, we define two new substitution models that are tailored to our bit-vector-based representation of cognate data. In Section 3.4, we introduce the data we use for our computational experiments. For comparing results inferred under different models, we use the AIC (Akaike Information Criterion) score (see Section 3.5). In Section 3.6 we present the results of our experiments. First, we analyze the parameter estimates resulting from the newly introduced models in ( Section 3.6.1), before we compare all models under study to each other (see Section 3.6.2). Our observations motivate a detailed examination of the properties of the input data ( Section 3.6.3) which indicate, that the newly introduced models might be overparametrized. We substantiate this assumption via a cross-validation study presented in Section 3.7. We conclude and discuss our results in Section 3.8.

### 3.1 Motivation

Cognate data are typically encoded via binary character matrices (see Section 3.2.1). Apart from the straight-forward dataset assembly process, a clear advantage of binary character matrices is that the corresponding evolutionary models only have but a few free parameters that need to be estimated. Therefore, these models can also be applied to small datasets without the risk of overparametrization. However, the binary representation of cognate data also exhibits drawbacks. Binary cognate character matrices are composed of columns, and, for the tree inference process using the phylogenetic likelihood function, we assume that these columns evolve independently. Yet, as single concepts are represented by groups of binary columns of varying size, the assumption of independent per-column evolution constitutes a potentially biased over-simplification of the process ( Evans *et al*., 2006). To illustrate this, let us consider two groups of eight columns each. The first group represents four different concepts with two cognate classes each, so that two columns belong to each concept. The second group represents a single concept with eight cognate classes. Further, let us assume that there is no missing information; that is, the data contain at least one word for each language and each concept. It follows that in the first group of columns, the value 1 occurs at least four times for each language. However, if the value 1 occurs four times in the second column group, this means that there exist words from four different cognate classes that describe the corresponding concept in the respective language. As the concepts used in these datasets are however typically fundamental ones, the occurrence of the above constellation is comparatively unlikely. Yet our example illustrates that the columns are clearly *not* independent of each other.

The standard binary encoding does also not allow to model unseen states at the ancestral nodes ( Evans *et al*., 2006) as there is one column per cognate class in the observed data. Beyond that, further cognate classes can only be accommodated if the binary model is combined with ascertainment bias correction ( Lewis, 2001).

( Evans *et al.*, 2006) also criticize that the underlying evolutionary model is time-reversible, that is, one assumes that evolution occurs in the same way if followed forward or backward in time. Non-time-reversible models are however more parameter rich and their application is numerically challenging ( Bettisworth & Stamatakis, 2020). Binary character matrices together with the accompanying models are hence an imperfect approximation for lexical evolution.

The aforementioned shortcomings of the binary encoding and binary models of character evolution raise the question if it is possible to represent cognate data in a different way. To this end, we introduce a bit-vector-based representation for cognate data where one site corresponds to exactly one concept (see Section 3.2.2) and we propose distinct evolutionary models tailored to this novel representation of cognate data (see Section 3.3).

The challenge we are facing is analogous to that of determining substitution rates (that form part of evolutionary models) for amino acid data. As amino acid data has 20 character states, the number of free parameters in the character substitution model (189 substitution rates) is typically also far too high for the rates to be reliably estimated on a single character matrix. Instead, models with a given, fixed substitution matrix are used. The substitution rates of these models are determined using large databases of amino acid data ( Tinh *et al*., 2024). *LG*, a widely used amino acid substitution matrix, is for example based on almost 4000 aligned amino acid sequences ( Le & Gascuel, 2008). The number of available cognate datasets is substantially smaller (see Section 2). Therefore, it is not possible to apply an analogous approach to cognate data with a large number of states.

### 3.2 Cognate data

Each cognate dataset is based on a list of concepts. Collecting data for the languages under study results in an assignment of a set of words to each language-concept pair. From these data, we construct a matrix *M* containing the words’ cognate classes. Cognate classes unite words that have been derived from a common ancestor ( Dunn, 2013) (see 3 (b)). Hence, *M* describes the following function:

M:(L×C)∧→V

$$(l,c)\;\wedge \mapsto V\subset V_{c}$$

where *L* contains the languages and *C* the concepts under study. For a concept *c* ∈ *C, V _c_* is the set of cognate classes comprising all words describing this specific concept *c* in the languages under study. We also denote the set *V*, which contains the cognate classes for a language-concept pair, as *state* in the following. We denote henceforth the size of *V _c_* by *κ* and the size of *V* by *ν*. The variable *κ* hence corresponds to the number of cognate classes that exist for a certain concept, while *ν* gives the number of cognate classes that are present for a language and a concept. We assume that the concept lists have been reasonably assembled, that is, there exists at least one word for each concept in each language. When *ν* = 0 we interpret this as missing information. You can find an overview over all important terms introduced in this section in Table 6.

**Figure 3.**
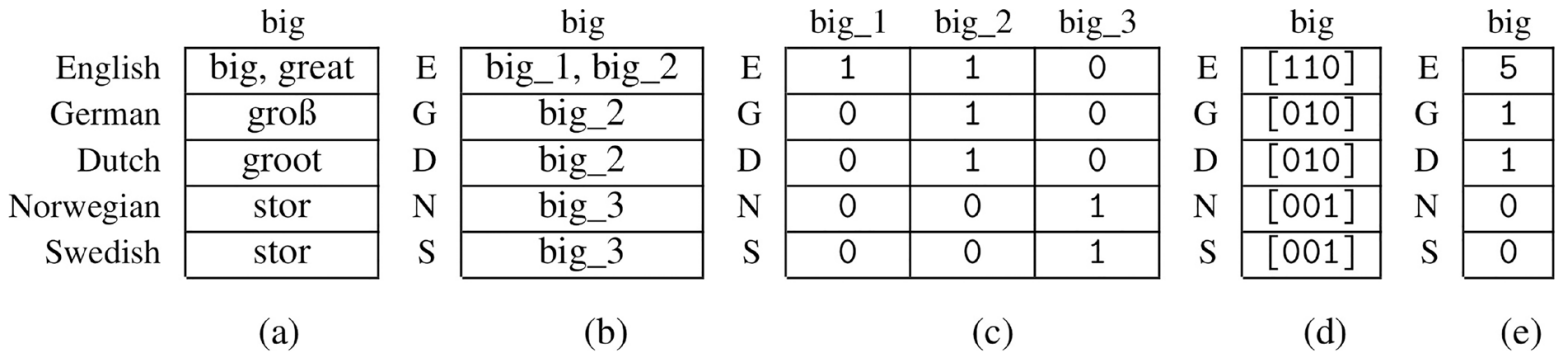
( **a**): Native cognate data ( **b**): Corresponding matrix *M* with cognate classes ( **c**): Binary character matrix *A ^b^* ( **d**): Matrix with bit vectors ( **e**): Character Matrix in the newly introduced format *A ^v^*.

***3.2.1 Binary representation***. A cognate dataset can be represented by a binary character matrix *A ^b^* containing the symbols 0 and 1. Additionally, specific entries may be set to the undetermined character -, to represent missing information. We obtain *A ^b^* as the presence-absence-matrix corresponding to the matrix containing the cognate classes (see Figure 3 (c)). Each concept is therefore represented by *κ* columns, where each column corresponds to a specific cognate class. If a certain cognate class is present in the state of a language, the respective entry is set to 1, and to 0, otherwise. Thereby, we assume that for each concept, there exists at least one word in every language. If there no cognate class is provided for a language and a concept, this will be interpreted and modeled as missing information. Consequently, we set all columns corresponding to this concept to -.

***3.2.2 Bit-vector-based representation***. We now introduce a new format for encoding cognate data for phylogenetic inference that relies on bit vectors. Let *A ^v^* be a character matrix for this newly introduced format. In analogy to the binary representation, *A ^v^* is based on the presence-absence matrix of the corresponding cognate dataset. However, unlike *A ^b^*, each concept *c* is only represented via a single column in *A ^v^*. This column conceptually contains the complete presence-absence bit vector for this concept *c* (see Figure 3 (d)). However, the ML-based tree inference tool RAxML-NG (see Section 3.4) cannot directly process character matrices where the entries are bit vectors. To circumvent this, we use a multi-valued encoding in *A ^v^*. In a multi-valued character matrix, each entry is a symbol from an ordered list *Σ _m_*. For each bit vector *b* we determine *∫*( *b*), that is, the integer with the binary representation *b*. Then, we use *∫*( *b*) as a pointer to *Σ _m_* to determine the symbol that corresponds to *b*. Note that the 0-bit vector never occurs. It would represent the case that there is no word provided for a language and a concept, which we however interpret as missing information. We can therefore skip 0-bit vector when assigning symbols to the bit vectors. Consequently, we subtract 1 from *∫*( *b*) when using it as a pointer to *Σ _m_*. Overall, we obtain the values in the column representing a concept *c* in *A ^v^* (see Figure 3 (e)) by applying the following function to the bit vectors:

$${\left\{0,1\right\}}^{\kappa }\wedge \to \Sigma_{m}$$

$$b\;\wedge \mapsto s=\Sigma_{m}\left[\int (b)-1\right]$$

The number of possible symbols for a column representing a concept *c* depends on *κ*. Therefore, in order to be able to conduct phylogenetic likelihood calculations, the datasets must be subdivided in such a way that each subset only contains concepts with exactly the same number of cognate classes. We henceforth call a subset of concepts that contains concept with exactly *κ* cognate classes a *κ*-subset. In RAxML-NG, multi-valued character alphabets are restricted to a maximum of 64 distinct symbols ( Kozlov *et al*., 2019). We require 2 *^κ^* – 1 symbols to represent a concept with *κ* cognate classes. Our approach is therefore currently limited to concepts with at most 6 cognate classes. Furthermore, we do not consider the case *κ* = 1. The corresponding concepts yield a single cognate class only, thus do not provide any signal for the resulting tree topology.

***3.2.3 Assumptions about the Evolution of Lexica***. Both the base frequency vector and the rate matrix (see 3.3) in our newly introduced models are highly symmetric. The symmetries are based on the following assumptions about the evolution of language lexica:

- (B1) For each language-concept-pair, it holds that *ν >* 0. In the case *ν =* 0, we assume missing data.
- (B2) The probability for a state occurring for a language-concept pair only depends on *ν*.
- (B3) The above probability decreases with increasing *ν* ( Levinson, 2000).
- (S1) At most one word can emerge or disappear within a single infinitesimal time step.
- (S2) The probability for a new word to emerge only depends on the value of *ν* in the current state.
- (S3) The above probability decreases with increasing *ν*.

The assumption (B1) is based on the fact that we assume missing data if there is no word provided for a language and a concept. Note that this is implicitly realized via the bit-vector-based representation, as there exists no symbol for encoding the 0-bit-vector. Assumptions (B2) and (B3) are based on an empirical study presented in Section 3.6.3. Assumptions (S1), (S2), and (S3) are not supported by any quantitative observations. Such evidence could be generated via independent estimates of substitution rates using a General Time Reversible model (see Section 3.3) and a subsequent analysis of these rates to determine the extent to which they are consistent with our assumptions (S1, S2, S3). However, obtaining meaningful estimates for such a large number of independent rates i.e., free parameters) requires an extremely large amount of input data that is simply not available at present. Instead, we analyze the consistency of assumptions (S1) and (S3) with the rates estimated by our newly introduced models (see Section 3.6.1).

### 3.3 Substitution models

Beyond adequately representing the input data, the selected substitution model can have a major impact on the resulting tree topology of a phylogenetic inference. Let us consider a character matrix containing *s _max_* symbols *Σ* = ( *Σ*[1],..., *Σ*[ *s _max_*]). We define a *substitution model* as a tuple ( *Q,R*) following the notation of Yang (2006). For *i* ∈ (1,…, *s _max_*), *Q*[ *i*] is the probability with which *Σ*[ *i*] initially occurs. We denote the probabilities in *Q* as *base frequencies*. For each *i,j* ∈ (1,…, *s _max_*), *R*[ *i*, *j*] provides the instantaneous rate of substitution between *Σ*[ *i*] and *Σ*[ *j*]. Since we model evolution as a continuous time Markov Chain, this value depends on nothing but *Σ*[ *i*]. Further, we assume that evolution is a time reversible process. Hence, it holds that *Q*[ *i*] *R*[ *i*, *j*] = *Q*[ *j*] *R*[ *j,i*] ∀ *i*, *j* ∈ (1,…, *s _max_*). For further details on these processes please consult the standard textbook by ( Yang, 2006). As the main subject of this work is the initial exploration of the newly introduced models, we do not model rate heterogeneity as this would introduce at least one additional free parameter.

For tree searches on binary character matrices, we use the *BIN* model of binary character substitution. This model is very simple and only has a single free parameter (a single base frequency). For multi-valued character matrices that result from our novel bit-vector-based representation of cognate data, we can deploy distinct models. Here, we investigate the most parameter-rich General Time Reversible ( *GTR*) model ( Tavaré, 1986) and the most parameter-poor *MK* model ( Lewis, 2001). In the GTR model, *all* substitution rates and base frequencies are estimated independently of each other. Therefore, the number of free parameters grows quadratically with the number of distinct symbols in the character matrix. For example, a GTR model for *κ*-subset of size three already has 36 free parameters. This induces increased runtimes and, more importantly, potential overparametrization, as we have to operate on small datasets. The MK model constitutes a viable alternative as it can potentially alleviate both aforementioned challenges. In this model, all substitution rates and all base frequencies are equal. Hence, the MK does not admit any free parameter. However, due to its simplicity, the model might tend not to be sufficiently flexible and to oversimplify the evolutionary process. In the following, we present two models, *COG* and *COGs* that are tailored to the bit-vector-based representation of cognate data. The aim is to determine a compromise between GTR and MK. To this end, we introduce symmetries for both, the base frequencies, and the substitution rates that represent the evolution of the lexica of languages. The resulting models have substantially fewer free parameters than GTR, but are more flexible than MK. An overview of the existing and newly introduced models is provided in Table 1.

**Table 1.** Models and their numbers of free parameters depending on *κ*.

Model	Number of free parameters ( *κ*)
BIN	1
GTR	$\frac{2^{\kappa }\cdot 2^{\kappa -1}}{2}+2^{\kappa }$
MK	0
COG	2 *κ*
COGs	*κ* + 1

***3.3.1 Symmetries for base frequencies***. Following Assumption (B2), the base frequency for a symbol in the character matrix *A ^v^* only depends on *ν*, that is, the size of the state it represents. Conversely, the base frequency is independent of the specific cognate classes, and thus of the position of the 1s in the bit vector. Let *s* be a symbol representing a bit vector *b*, which encodes a state of size *ν*. Note that *ν* corresponds to *popcnt*( *b*), which we define as the number of 1s in *b*. For the base frequencies it hence results that *Q*[ *s*] = *π _ν_*. In the case of *κ* = 3 *Q* looks as illustrated in Figure 4a.

**Figure 4a.**
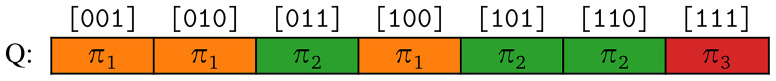
Symmetries of base frequencies for *κ* = 3 in the models COG and COGs.

According to assumption (B3), we expect *π _v_* to decrease with *v*. We analyze this in Section 3.6.1.

***3.3.2 Symmetries for substitution rates***. We develop two different evolutionary models for the bit-vector-based representation. The two models differ with respect to the symmetries of the substitution rates. *COGs* is the simpler model as it only has two free parameters. In the following, we use it to examine the correctness of assumption (S1). *COG* is more complex as the corresponding rate matrix contains symmetries that reflect assumptions (S1) and (S2).

**3.3.2.1 COGs**

The rate matrix of the model COGs is highly symmetrical, which results in two distinct rates only. The fist rate *λ*_+_ applies to all symbol pairs (transitions) where the corresponding states differ only by the presence or absence of one cognate class. The second rate *λ*_0_ holds for all remaining transitions that differ by presence or absence of strictly *more* than one cognate class. Let *b*_1_, *b*_2_, *b*_1_ ≠ *b*_2_ be bit vectors and *s*_1_ = *Σ _m_* [∫( *b*_1_)–1], *s*_2_ = *Σ _m_* [ *∫*( *b*_2_)–1] the corresponding symbols. The respective substitution rate belongs to the first group if and only if *popcnt*( *b*_1_ ⊕ *b*_2_) = 1. In the rate matrix for the COGs model, we hence set *R*[ *s*_1_, *s*_2_] = *λ*_+_ if *popcnt*( *b*_1_ ⊕ *b*_2_ ) = 1 and *R*[ *s*_1_, *s*_2_] = *λ*_0_ if *popcnt*( *b*_1_ ⊕ *b*_2_) > 1. The symmetries resulting for *κ* = 3 are illustrated in Figure 4b.

**Figure 4b.**
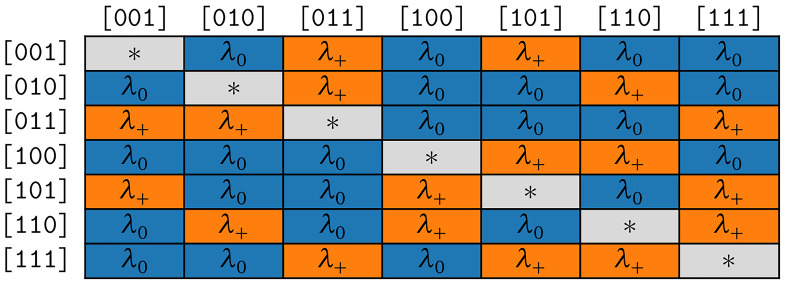
Symmetries of substitution rates for *κ* = 3 in the model COGs.

According to assumption (S1), we expect *λ*_0_ to be close to 0. We analyze the corresponding empirical maximum likelihood estimates in detail in Section 3.6.1.

**3.3.2.2 COG**

The COG model has a rate matrix with symmetries that reflect assumptions (S1) and (S2). As in COG, there is a transition rate *λ*_0_ that applies to all pairs of symbols whose states differ by the presence or absence of strictly more than one cognate class. In contrast to COGs, this rate is forced to be 0 in COG in order to comply with assumption (S1). Let *b*_1_, *b*_2_, *b*_1_ ≠ *b*_2_ be bit vectors and *s*_1_ = *Σ _m_* [ *∫*( *b*_1_)–1], *s*_2_ = *Σ _m_* [ *∫*( *b*_2_)–1] the corresponding symbols. We set *R*[ *s*_1_, *s*_2_] = *λ*_0_ if if *popcnt*( *b*_1_ ⊕ *b*_2_) > 1, forcing *λ*_0_ = 0. In the case that the corresponding states for a pair of symbols differ in the presence or absence of only one single cognate class, we allow for different rates depending on the size *ν* of the smaller one of the two states. As *ν* is equal to the population count (that is the number of bits set to 1) in the corresponding bit vector, we set *R*[ *s*_1_, *s*_2_] = *λ*_ν_ with *ν* = *min*( *popcnt*( *b _i_*), *popcnt*( *b _j_*)) if *popcnt*( *b*_1_ ⊕ *b*_2_) = 1. Thereby, we implement assumption (S2) in the COG model.

The symmetries resulting for *κ* = 3 are depicted in Figure 4c.

**Figure 4c.**
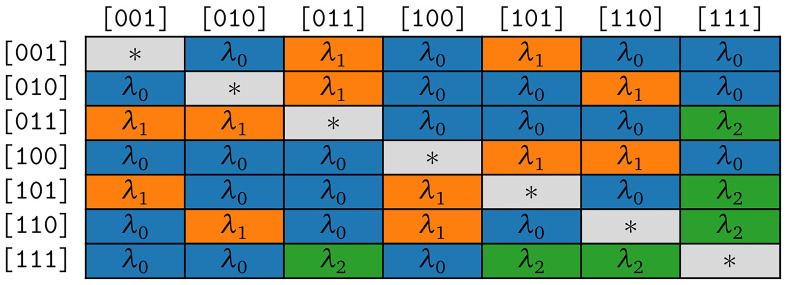
Symmetries of substitution rates for *κ* = 3 in the model COG.

According to assumption (S3), we expect the estimates for *λ*_ν_ to decrease as *ν* increases. We analyze this in Section 3.6.1.

### 3.4 Experimental setup

Our experiments are based on the cognate datasets from the *Lexibench* database ( Häuser & List, 2025) which we access via *PyLexibank* ( List *et al.,* 2022; List *et al.,* 2023). We access via *PyLexibench* ( Häuser *et al*., 2025a and Häuser *et al*., 2025b [Software]) to obtain the corresponding character matrices. For each dataset, we extract the *κ*-subsets for *κ* ∈ [2,6]. (see Section 3.4.1 *κ*-subset under study, we construct both, the binary, and bit-vector-based character matrix as described above. The corresponding source code is included to PyLexibench. We implemented both COGs and COG in RAxML-NG ( Häuser, 2025d [Software]). Our experiments are documented and available online ( Häuser, 2025b [Code]). For each *κ*-subset, we perform five separate analyses, that is, one under each of model: BIN, MK, GTR, COGs, and COG. The inferences under BIN are performed on the binary character matrices. For the inferences under the remaining models we use the bit-vector-based representation. Each analysis comprises 20 independent maximum likelihood (ML) tree searches. To this end, we use the default tree search configuration of RAxML-NG (10 searches starting from random trees and 10 searches starting from randomized stepwise addition order parsimony trees).

**Figure 5.**
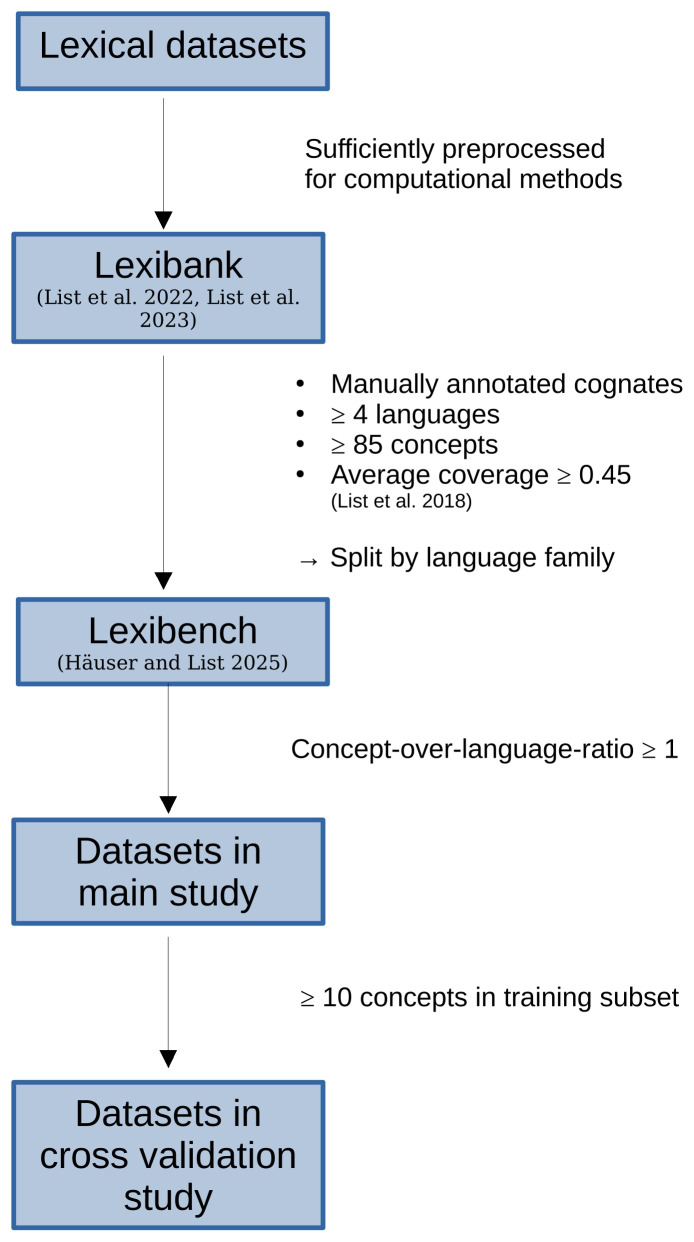
Data selection criteria. For our experiments, we use cognate datasets from Lexibench ( Häuser & List, 2025) which which is a subset of Lexibank ( List *et al.,* 2022; List *et al.,* 2023).

***3.4.1 Data Inclusion Criteria*.** In the following we explain in more detail, how we select the input data for our experiments. This process is also illustrated in Figure 5.

All datasets under study are part of the *Lexibank* ( List *et al.,* 2022; List *et al.,* 2023) lexical database that contains all published lexical data sets that have been sufficiently preprocessed to be used as input for computer-based methods.

The *Lexibench* benchmark, consists of those Lexibank datasets that cover at least 4 languages and 85 concepts and for which manually annotated cognates are available ( Häuser & List, 2025). In addition, they must exhibit an average coverage ( List *et al.,* 2018) ≥ 0.45. The datasets in Lexibench are further subdivided according to the languages families they comprise.

For our main study, we use the *κ*-subsets of the Lexibench datasets with a language-over-concept-ratio ≥1, that is, those that comprise at least as many concepts as languages. For specific *κ* values, our experiments do therefore not include all Lexibench datasets.

In order to be used in the cross-validation study (see Section 3.7), the datasets must additionally yield a training subset with at least 10 concepts.

The 20 independent tree searches for a given subset can potentially lead to different parameter estimates and a different final log likelihood score. In the following, we only consider the estimates resulting from the tree search that yields the best final log likelihood score.

We further filter the Lexibench datasets in order to obtain those that are suitable for our studies (see also Section 3.4, Section 3.7.1).

### 3.5 Comparing models

To compare different models, we deploy the Akaike Information Criterion (AIC) score ( Akaike, 1998). In phylogenetics, this criterion provides an estimate of the information, which is lost when choosing a specific model to represent the evolutionary process. To minimize this loss, a model must neither be too simple nor too complex. A lower AIC score indicates that the result obtained with the respective model is superior. However, the AIC score is only suitable for comparing the results of inferences conducted on the same input data, that is, data represented in the same manner. Hence, the AIC scores resulting for the inferences executed under the BIN model on the binary character matrices cannot be compared to those obtained for the inferences under the remaining models on the bit-vector-based character matrices.

### 3.6 Results

In the following, we present the results of our study. First, we consider the rates estimated under the newly introduced models and examine to which extent our observations reflect our assumptions about the evolution of the language lexica. Then we compare the different bit-vector-based models via AIC scores. In Section 3.6.3 we additionally present a detailed analysis of specific properties of the input data.

***3.6.1 Frequency and rate estimates***. Subsequently, we analyze the estimates of the base frequencies and substitution rates obtained from the inferences with the newly introduced models COGs and COG on the *κ*-subsets for the input data. We restrict ourselves to *κ* = 3 here. For *κ* = 2, the rate matrix is too small to draw meaningful conclusions. For *κ* > 3, we are on the verge of overparameterization so that conclusions drawn from the estimated rates are also less meaningful (see Section 3.7 and Section 3.6.3).

**3.6.1.1 COGs**

**Figure 6.**
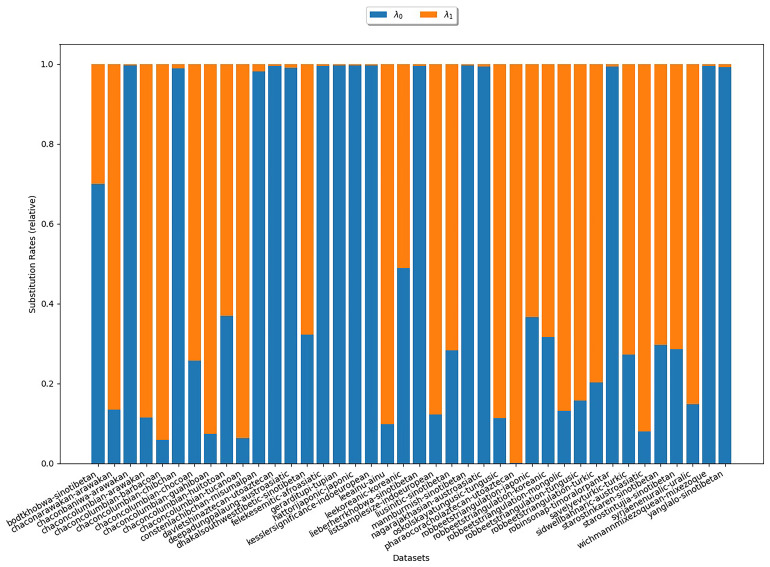
The plot illustrates the substitution rates estimated with COGs for *κ* = 3. Each bar represents an analyzed *κ*-subset. The heights of the differently colored areas correspond to the substitution rates relative to each other. There is no clear trend as to which of the two rates is higher.

Initially, we consider the substitution rates estimated with COGs. We are particularly interested in the estimates for the rate λ _0_. If they come close to 0, this substantiates assumption (S1). Substitution rate estimates for the *κ*-subsets ( *κ* = 3) are depicted in Figure 6. In the plot, there is one bar for each *κ*-subset under study. The heights of the different colored areas correspond to the substitution rates relative to each other. There is no clear trend as to which of the two rates is higher. In particular, this experiment does not reflect assumption (S1).

**3.6.1.2 COG**

We further analyze the estimates obtained from the inferences under the COG model. Here we aim to examine, whether the base frequencies *π _ν_* decrease with growing *ν*, which would substantiate assumption (B3).

**Figure 7.**
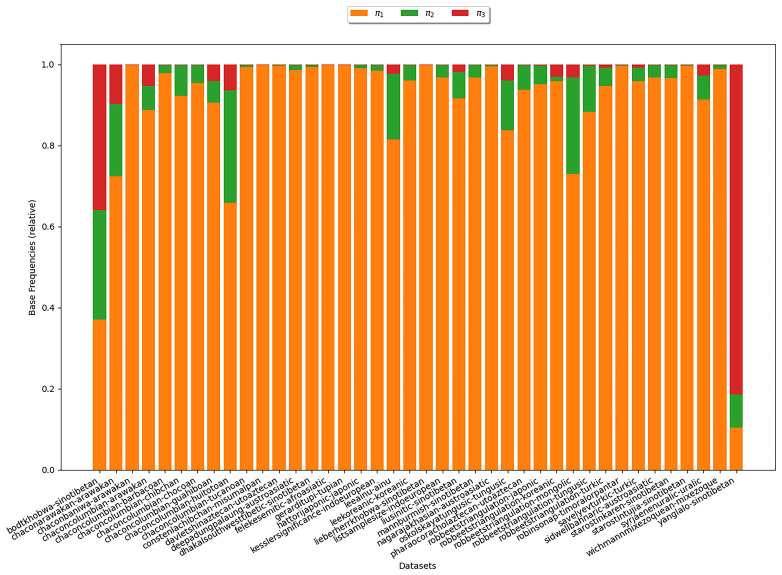
The plot shows the base frequencies estimated under COG for *κ* = 3. Each bar represents a *κ*-subset. The heights of the differently colored areas correspond to the base frequencies relative to each other. We observe that for most datasets, *π*_1_ is largest indicating that symbols representing states with one cognate class yield the highest base frequency.

Figure 7 illustrates the estimates for the base frequencies for *κ*-subsets ( *κ* = 3). In the plot, there is one bar for each *κ*-subset under study. The heights of the different colored areas correspond to the base frequencies relative to each other. We observe that, for most datasets, *π*_1_ is largest. That is, symbols representing states with one cognate class have the highest base frequency. This is in line with the empirical observation illustrated in section 3.6.3 and therefore provides additional evidence that assumption (B3) is realistic.

**Figure 8.**
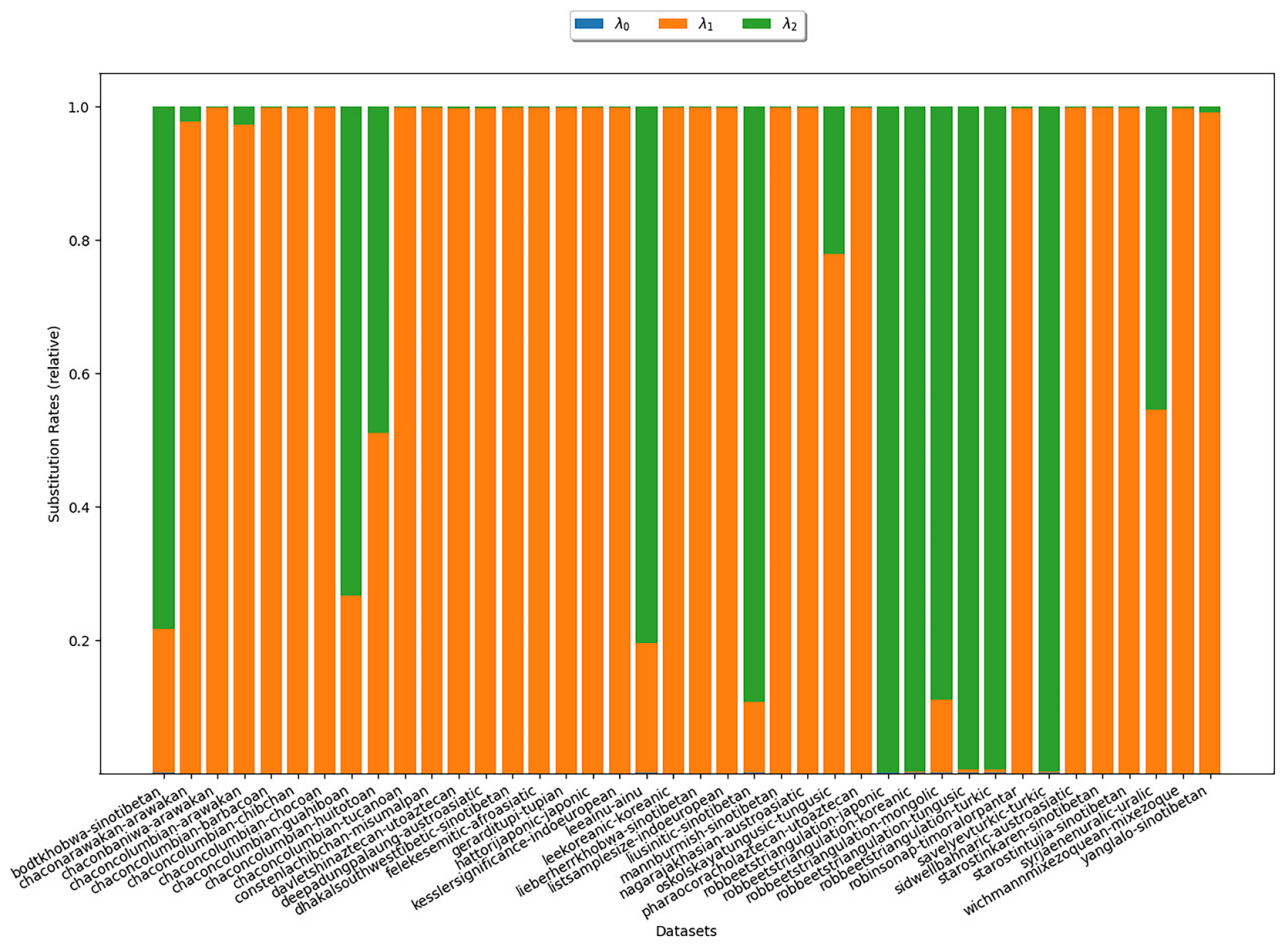
The plot shows substitution rates estimated under COG for *κ* = 3. Each bar represents a *κ*-subset. The heights of the differently colored areas correspond to the substitution rates relative to each other. For most datasets we observe that *λ*_1_ > *λ*_2_, which is in line with assumption (S3).

Analogously, we investigate whether the rates *λ _ν_* decrease with growing *ν*, which could substantiate assumption (S3). Substitution rate estimates for *κ*-subsets ( *κ* = 3) are shown in Figure 8. In the plot, there is one bar for each *κ*-subset under study. The heights of the differently colored areas correspond to the substitution rates relative to each other. For all datasets it holds that *λ*_0_ = 0. This merely demonstrates the correctness of our implementation. For > 70% of the datasets, we observe that *λ*_1_ > *λ*_2_, which is in line with assumption (S3).

**Figure 9.**
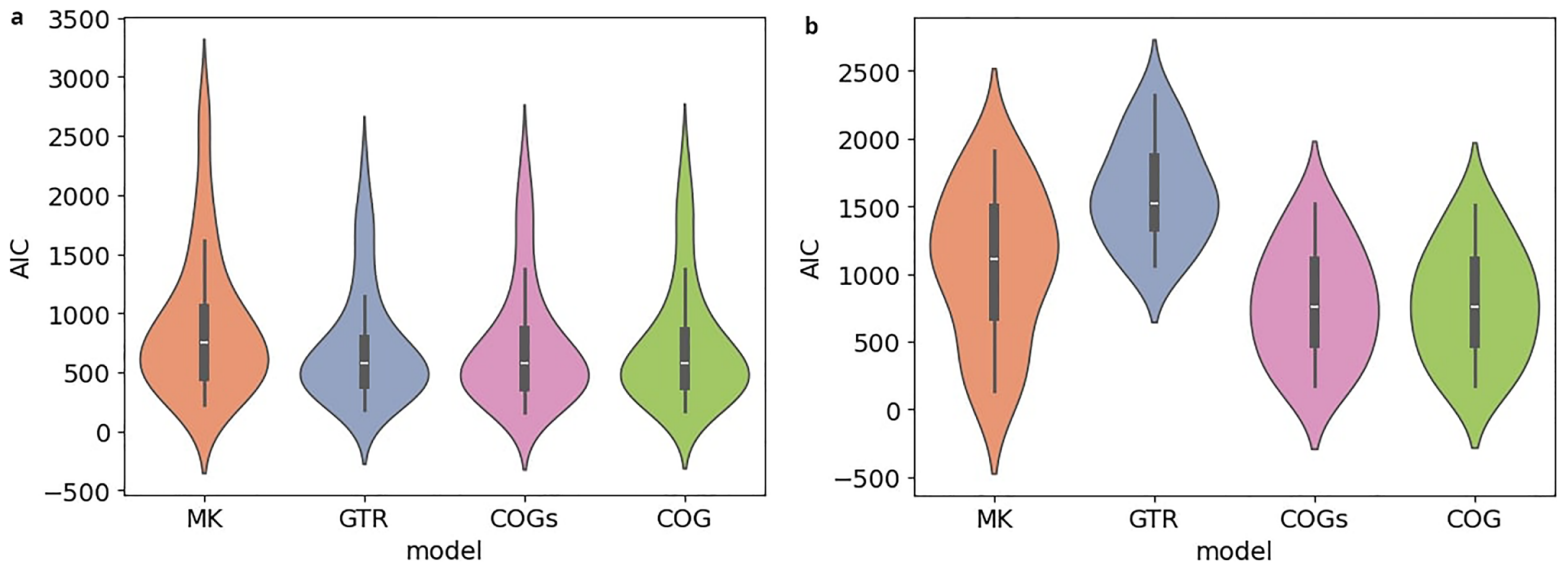
The figures compare the AIC scores obtained under different models for *κ*-subsets with a size of *κ* = 3 ( **a**) and of *κ* = 5 ( **b**). The y-axis indicates the AIC score. For each model, a violin plot illustrates the distribution of AIC scores for the *κ*-subsets under study. For *κ* = 3, the models fit the data comparatively well. For *κ* = 5, GTR yields a substantially poorer model fit, presumably due to the quadratically increasing number of free parameters.

***3.6.2 Model comparison***. In the subsequent analysis, we compare the AIC scores for the different models. In Figure 9 (a), the results for *κ* = 3, in Figure 9 (b) those for *κ* = 5 are shown. Both plots are structured in the same way, with the y-axis indicating the AIC score. For each model, there is a violin plot illustrating the distribution of AIC scores for the *κ*-subsets under study. Inferences under the BIN model are performed on binary rather than bit-vector-based character matrices. Thus, we restrain our analysis to the four remaining models. Recall that a lower AIC score indicates a better model fit. The analysis of the scores thus reveals that for *κ* = 3, the models fit the data comparatively well. For *κ* = 5, GTR yields a substantially poorer model fit, which is presumably due to the quadratically increasing number of free parameters. The AIC scores obtained for five exemplary datasets (see Table 2) are in line with what we observe in the plots.

**Table 2.** AIC scores obtained under different models for the *κ* = 3- and *κ* = 5-subsets of five exemplary datasets. The full table is published online ( Häuser, 2025e [Data]).

	*κ* = 3				*κ* = 5			
dataset	MK	GTR	COGs	COG	MK	GTR	COGs	COG
chaconbaniwa-arawakan	1382	1006	976	980	1164	1655	758	762
chaconcolumbian-huitotoan	404	413	391	405	138	1062	185	181
liusinitic-sinotibetan	806	708	728	730	1603	2051	1270	1274
oskolskayatungusic-tungusic	1158	1011	1039	1057	1812	2208	1470	1463
robbeetstriangulation-tungusic	1536	1365	1375	1376	1910	2318	1519	1513

***3.6.3 Properties of cognate data***. In the following, we examine the available cognate datasets with regard to some specific properties. The observations allow to explain the behavior of both the established and the newly introduced models.

**3.6.3.1 Distribution of *ν***

**Figure 10.**
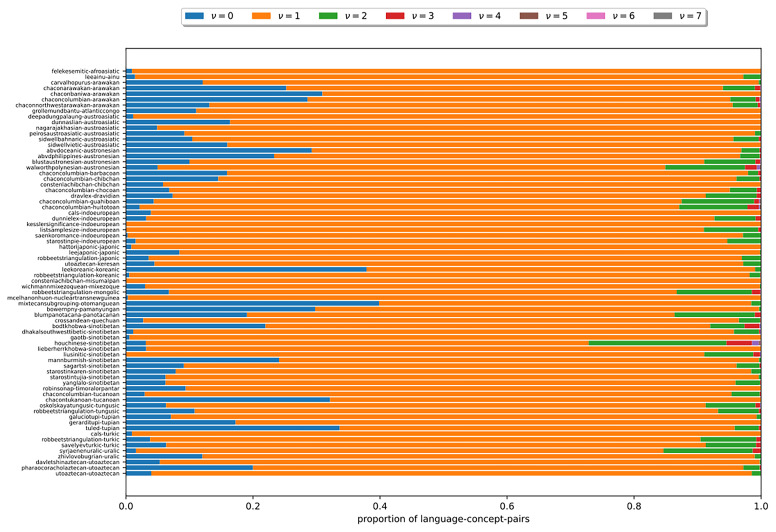
Each bar in this figure corresponds to a dataset (see Section 3.4). The y-axis indicates the proportion of language-concept pairs in the respective dataset. The differently colored segments correspond to different numbers of *ν*. The figure shows that the higher *ν* is, the fewer language-concept pairs with the corresponding number of cognate classes in their state occur.

In the following, we examine, how *ν*, the number of cognate classes existing per language-concept pair, is distributed in the datasets under study. In Figure 10, each bar corresponds to a dataset. The y-axis indicates the language-concept pair proportion in the respective dataset. The differently colored segments correspond to different values of *ν* that depict the varying number of cognate classes in the states of the language-concept pairs. The figure shows that across all datasets, it holds that *ν* = 1 for most language-concept pairs. The higher *ν*, the fewer language-concept pairs with the corresponding number of cognate classes in their state occur. Furthermore, the results show that some datasets comprise a substantial amount of missing data ( *ν* = 0). The observation substantiate assumptions (B2) and (B3). It also illustrates that the datasets exhibit a low degree of polymorphism. Nevertheless, polymorphic entries should not be ignored due to their impact on the results of the tree inferences, as shown in ( Häuser *et al*., 2024a).

**3.6.3.2 Symbol frequencies**

**Figure 11.**
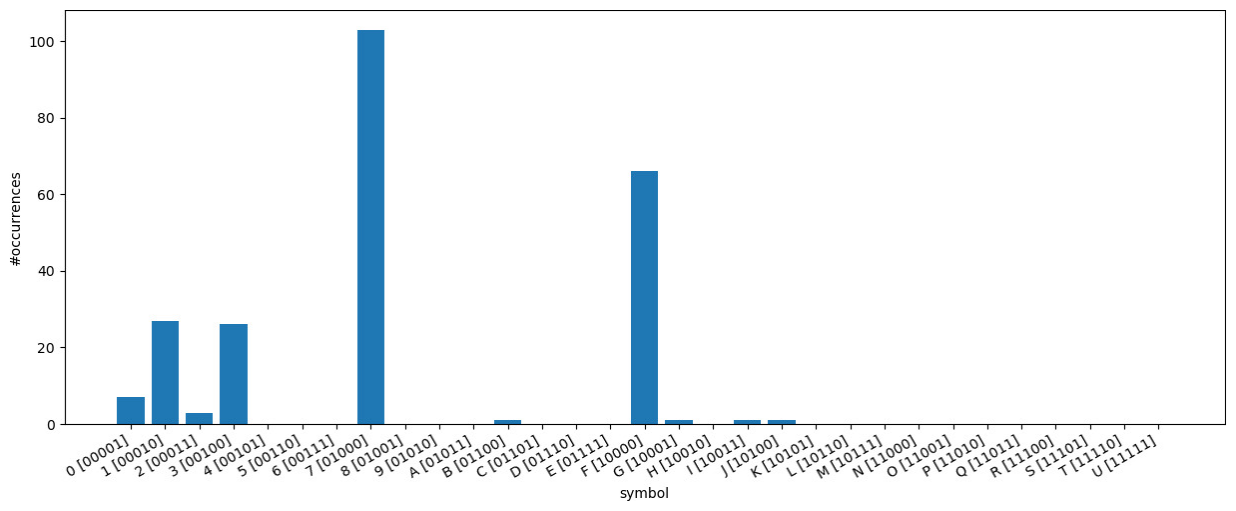
The figure illustrates how often each symbol appears in the *κ*-subset ( *κ* = 5) of the dataset *dunnielex-indoeuropean* ( Dunn, 2012). Each bar corresponds to a bit vector represented by a specific symbol in the character matrix. The y-axis indicates how often the respective symbol occurs in the character matrix. Symbols representing bit vectors with a single 1 occur very frequently. For many bit vectors with higher numbers of 1s, the corresponding symbols do not appear at all.

The observed distribution of *ν* has a direct impact on the symbol frequencies in the bit-vector-based character matrices, which we examine in the following. Figure 11 illustrates how often each symbol appears in the *κ* = 5-subset of the dataset *dunnielex-indoeuropean* ( Dunn, 2012). In this plot, each bar corresponds to a bit vector represented by a specific symbol in the character matrix. The y-axis indicates how often the respective symbol occurs in the character matrix. We observe that, a few symbols occur very frequently, while a large proportion of symbols does not appear at all. The most frequently occurring symbols are those that represent bit vectors with only a single 1. For many bit vectors where the number of 1s exceeds 1 or even 2, the corresponding symbols are not present in the character matrix, which follows from the observation presented in Figure 10. The analysis of the symbol counts further illustrates that some of the models examined are overparameterized. For GTR, this is obvious as it is not possible to reliably estimate the base frequencies or substitution rates for symbols that do not occur at all in the data. However, the COG model can also suffer from overparameterization. The symmetries introduced in this model are based on grouping the symbols according to the number of 1s in the corresponding bit vectors. However, all symbols that occur frequently correspond to bit vectors with a single 1. They all provide a signal for estimating the base frequency *π*_1_ and the substitution rate *λ*_1_. The other rates and base frequencies are estimated on the basis of the symbols that represent bit vectors with a higher number of 1s. However, these rarely or never occur. Therefore, reasonable or numerically stable estimates for the respective base frequencies and substitution rates are not to be expected. For other datasets and *κ*-subsets with *κ* ≠ 5 we make similar observations, indicating that overparameterization needs to be further investigated. To this end, we perform the cross-validation study described in section 3.7.

**3.6.3.3 *κ*-Subset sizes**

**Figure 12.**
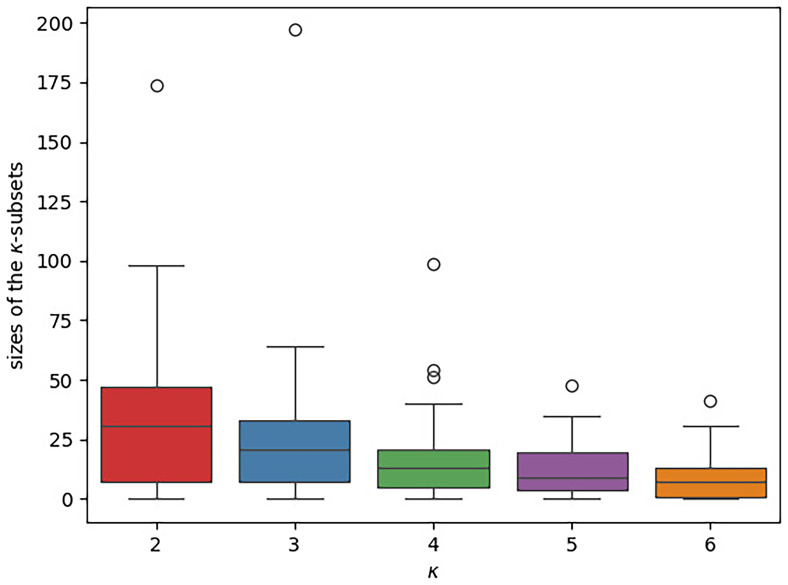
The boxplot illustrates the distribution of the different *κ*-subset sizes across the datasets under study. There is a separate box for each *κ* value. The *κ*-subset size, that is, the number of concepts a subset contains, is given on the y-axis. In each box, the median is indicated via a horizontal line. The *κ*-subsets are generally small (the vast majority comprises ≤ 50 concepts) and their sizes tends to further decrease with increasing *κ*.

The boxplots in Figure 12 illustrate the distribution of the *κ*-subsets sizes across the datasets under study. Refer to Table 3 for the exact numbers for five exemplary datasets. In the plot, there is a separate box for each *κ* value. The y-axis indicates the respective *κ*-subset size, that is, the number of concepts a subset contains. Overall, the subsets are small. Almost all subsets contain less than 50 concepts, a large part of them even less than 20. We further note that the size of the *κ*-subsets tends to decrease with increasing *κ*. In contrast to this, the number of free parameters in our newly introduced models increases with *κ*. Thus, the risk of overparameterization is highly likely to increase with *κ*.

**Table 3.** Number of concepts in the *κ*-subsets of five exemplary datasets. The full table is published online ( Häuser, 2025e [Data]).

dataset	*κ* = 2	*κ* = 3	*κ* = 4	*κ* = 5	*κ* = 6
chaconbaniwa-arawakan	66	64	33	21	10
chaconcolumbian-huitotoan	56	29	5	5	1
liusinitic-sinotibetan	36	29	14	22	13
oskolskayatungusic-tungusic	57	32	29	25	25
robbeetstriangulation-tungusic	57	40	30	24	21

**3.6.3.4 Concepts-over-languages-ratio**

**Figure 13.**
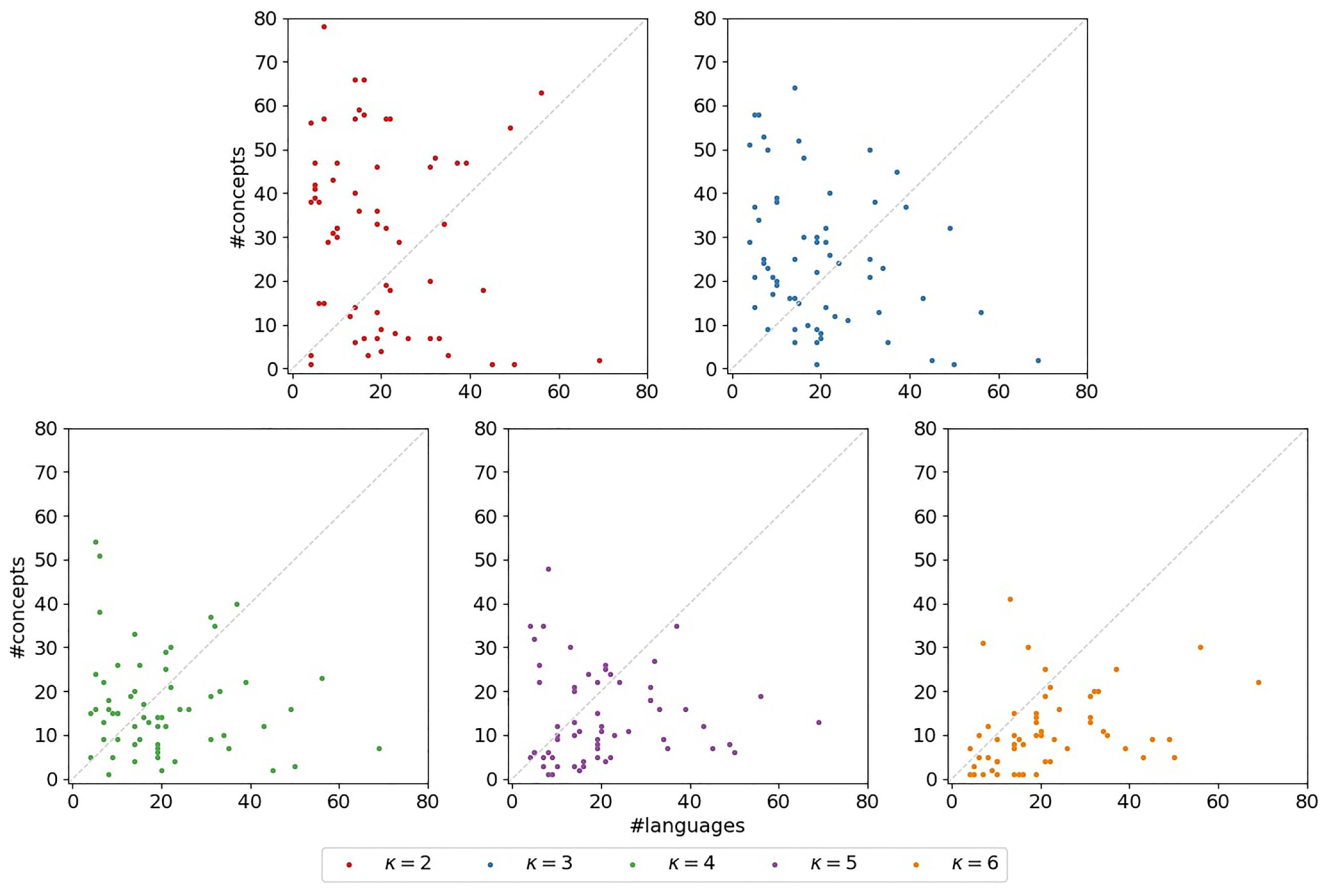
The plots illustrate the concepts-over-languages-ratios for the *κ*-subsets. There is one subplot for each *κ* ∈ [2,6]. In each of the subplots, the x-axis indicates the number of languages, the y-axis the number of concepts. Each marker corresponds to a *κ*-subset. The identity is indicated by a dashed line. If a marker is located below this identity, the corresponding subset exhibits fewer concepts than languages, which indicates a very poor signal.

To capture the shape of a *κ*-subset, we consider the *concepts-over-languages-ratio*, that is, the ratio between the number of concepts and the number of languages in the subset. This ratio corresponds to the ratio of the number of columns and the number of rows in the bit-vector-based character matrix representing the subset. A lower ratio can indicate a poorer the signal in the dataset. The scatter plots in Figure 13 illustrate the concepts-over-languages-ratios for the *κ*-subsets ( *κ* ∈ [2,6]) for the datasets under study. There is one subplot for each *κ* ∈ [2,6]. In each of the subplots, the x-axis indicates the number of languages, the y-axis the number of concepts. Each marker corresponds to a *κ*-subset. The identity is indicated by a dashed line. If a marker is located below this identity, the corresponding subset exhibits fewer concepts than languages, which indicates a very poor signal. We can observe that such *κ*-subsets occur for ∀ *κ* ∈ [2,6], but are more pronounced for increasing *κ*. In Table 4 we also observe poor concepts-over-languages-ratios for the exemplary *κ*-subsets, especially for higher values of *κ*. This is disadvantageous for our newly introduced models. With *κ*, the number of free parameters increases so that even larger datasets are necessary to obtain reasonable parameter estimates. These observations underline again the hypothesis that the models are overparameterized.

**Table 4.** Concepts-over-languages-ratio of the *κ*-subsets of five exemplary datasets. The full table is published online ( Häuser, 2025e [Data]).

dataset	*κ* = 2	*κ* = 3	*κ* = 4	*κ* = 5	*κ* = 6
chaconbaniwa-arawakan	4.71429	4.57143	2.35714	1.5	0.71429
chaconcolumbian-huitotoan	14	7.25	1.25	1.25	0.25
liusinitic-sinotibetan	1.89474	1.52632	0.73684	1.15789	0.68421
oskolskayatungusic-tungusic	2.71429	1.52381	1.38095	1.19048	1.19048
robbeetstriangulation-tungusic	2.59091	1.81818	1.36364	1.09091	0.95455

**3.6.3.5 Conclusion**

In this subsection, we examined the publicly available cognate data with respect to a plethora of numerical properties. We observed that for the vast majority of language-concept-pairs exactly 1 cognate class is provided. This is consistent with our assumptions about the evolution of lexica. Consequently, the different symbols in the bit-vector-based character matrices occur with highly dispersed frequencies, while a large proportion of these symbols are not present at all. Furthermore, we investigated the size and shape of the *κ*-subsets. We found that they are small and exhibit an insufficiently low concepts to languages ratio. Overall, these observations indicate that the parameter-rich models for the bit-vector-based representation might be overparametrized. We prove this by the cross-valdation study described in section 3.7.

### 3.7 Cross-validation study

The studies presented in the previous section 3.6.3 indicate that the examined *κ*-subsets are small and that the different symbols occur with distinct frequencies in the bit-vector-based character matrices. These observations indicate that the newly introduced COG model might be overparametrized. In the following, we conduct a cross-validation study to investigate this in greater detail.

***3.7.1 Experimental setup***. For our cross-validation study, we again restrict ourselves to the case of *κ* = 3. We randomly split each *κ*-subset into a training and a testing dataset. Eventually, we strive to detect overparameterization effects for the inferences on the complete *κ*-subsets. The larger the proportion of data used for training, the more realistic the approximation of inference behavior on the actual data will be. However, using a larger proportion of data for training induces smaller testing subsets. We hence exclude *κ*-subsets for which the corresponding bit-vector-based character matrices comprise less than 10 columns, as these are too small for conducting meaningful experiments (see Section 3.4.1).

When splitting the data in a 60: 40 ratio, there are *κ* = 3-subsets which are suitable as cross-validation input. For the remaining *κ* = 3-subsets, the testing subsets become too small. Subsequently, we construct binary and bit-vector-based character matrices for both, the testing, and the training subsets. On each training character matrix, we perform an inference under each of the five presented models using the standard configuration of RAxML-NG (see section 3.4). Let *llh _train_* be the best log likelihood score of the tree inferred under a particular model on the training data. In the following, we use this tree and the corresponding model parameter estimates resulting from this best scoring tree inference. Based on this best tree and model estimate, we evaluate the log likelihood for the testing subset, which we denote by *llh _test_*. Since training and testing subsets are of different size, we normalize the log likelihoods by the number of columns of the respective character matrix. We denote the relative likelihoods by $llh_{\text{train}}^{\text{rel}}$ and $llh_{\text{test}}^{\text{rel}}$ , respectively. We determine the relative error as


e:=llhtestrel−llhtrainrelllhtrainrel

For each *κ*-subset, we perform this procedure of random splitting 10 times (10-fold split) and compute the average error.

We further compare the degree of overparameterization in the *κ* = 3 subsets with that in the complete datasets that are the datasets in the standard binary representation without subdividing them into *κ*-subsets. To this end, we repeat the above experiment for the corresponding character matrices under the BIN model.

**Figure 14.**
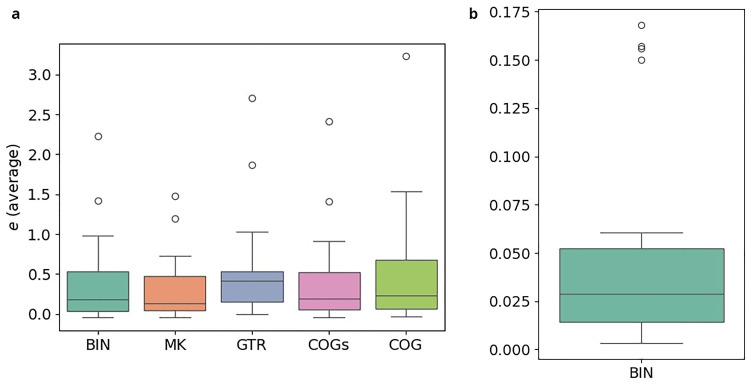
The plots illustrate the results of the cross-validation study. In both plots, the y-axis indicates the relative error *e* averaged over the 10-fold split. However, note that the scales between the left and the right plot differ substantially. For each model, there is one boxplot showing the distribution of the average relative error. The errors are at least 7 times higher when using the *κ*-subsets instead of the full datasets. ( **a**) Experiments on *κ* = 3-subsets ( **b**) Experiments on full datasets.

***3.7.2 Results***. The results of the cross-validation-study are illustrated in Figure 14. The exact numbers for five representative datasets are given in Table 5. Figure 14 (a) refers to the experiments conducted with the *κ* = 3-subsets, Figure 14 (b) to those with the standard binary datasets under the BIN model. In both plots, the y-axis indicates the relative error *e* averaged over the 10-fold split. Note, however, that the scales differ. For each model, there is one boxplot showing the distribution of the average relative error for the *κ*-subsets and the complete datasets, respectively. Using the *κ*-subsets, the median, indicated by a horizontal line in the boxplots, approximately ranges from 0.21 (MK) to 0.40 (GTR). This indicates substantial instabilities for all models. In contrast to that, we observe a median of 0.03 for the complete standard binary datatsets under the BIN model. The errors are thus at least 7 times higher when using the *κ*-subsets. It should be emphasized that this observation also holds for the MK model which does not have *any* free parameters. This indicates that the *κ*-subsets are too small to obtain stable inferences. Hence, applying these new models is not feasible, as they rely on subdividing the datasets into the *κ*-subsets. As the errors are high across all examined models, it is likely that other approaches based on subdividing the datasets will suffer from overparametrization as well. We conclude by observing that COG induces the lowest average errors among all more complex models we examined, which indicates that this model might be worth further consideration, provided that more data become available.

**Table 5.** Results of the cross-validation study for five exemplary datasets. For each model and each dataset the table provides the relative error *e* averaged over the 10-fold split. The results in the last column are obtained from experiments on the full datasets while all remaining results are based on the *κ*-subsets. The full table is published online ( Häuser, 2025e [Data]).

dataset	BIN	MK	GTR	COGs	COG	BIN (full)
chaconbaniwa-arawakan	0.0934	0.0948	0.1086	0.1009	0.1011	0.0186
chaconcolumbian-huitotoan	-0.0464	-0.0379	0.3408	0.0149	0.0677	0.0379
liusinitic-sinotibetan	0.4089	0.2841	0.4707	0.3220	0.3398	0.0129
oskolskayatungusic-tungusic	0.2154	0.1309	0.2529	0.1998	0.2171	0.0259
robbeetstriangulation-tungusic	0.3521	0.3233	0.4975	0.3839	0.4653	0.0297

**Table 6.** Glossary for Section 3.

*κ*	Number of cognate classes existing for a concept in a cognate dataset
*v*	Number of cognate classes present for a language and a concept in a cognate dataset
*A ^b^*	Binary character matrix
*A ^v^*	Character matrix in bit-vector-based representation
*κ*-subset	Subset of a cognate dataset containing only concepts with *κ* cognate classes
*e*	Relative error at cross-validation

### 3.8. Conclusion and discussion

In this section, we introduced a bit-vector-based representation of cognate data (see section 3.2.2). In the corresponding character matrices, there exists exactly one column for each concept of the corresponding cognate dataset. Thereby we aim to alleviate the drawbacks of the standard binary representation. We further show how one can subdivide the input datasets into so-called *κ*-subsets that are necessary for conducting likelihood-based phylogenetic inferences on bit-vector-based character matrices. We also introduced specific assumptions about the evolution of lexica. Based on these assumptions, we devised the COGs and COG models that are tailored to the bit-vector-based character matrices (see section 3.3). Although the datasets’ properties (see section 3.6.3) already indicate that the models we introduced might be overparametrized, we thoroughly assess the results of the inferences under these models. More specifically, we examine the transition rates estimated by respective phylogenetic inferences on the *κ* = 3-subsets under our new models (see Section 3.6.1). The estimated rates at least partially support our assumptions about the evolution of lexica. In addition, we compare different substitution models based on their AIC scores. We find that for higher values of *κ*, our new models outperform the GTR model. To properly investigate apparent overparameterization, we also conduct a cross-validation-study (see Section 3.7). We find that for *κ* = 3, substantial instabilities occur across all models, even under the MK model, which does not exhibit any free parameters. This illustrates that our models are currently inapplicable as they require subdividing the dataset into potentially too small subsets for which stable parameter estimates can not be obtained. However, inferences under the COG model still appear to be the most stable among all models under study. In conjunction with the result from the AIC scores analysis, this suggests that the COG model merits further investigation should larger cognate datasets become available. As long as this is not the case, we recommend using the standard and less parameter-rich models and dataset representations instead. Our results indicate that it is likely that any model, which is more parameter-rich and/or based on subdividing the dataset will suffer from overparametrization, given the currently available data.

## 4 Application of machine learning methods

Another motivation for assembling more linguistic datasets is the application of machine learning methods. Many recent advances in the field of phylogenetics are based on such approaches ( Azouri *et al.*, 2021; Haag *et al.*, 2022; Nesterenko *et al.*, 2024; Trost *et al.*, 2024). In the following, we examine how and more importantly *if* the use of such tools can currently be extended to cognate data.

To this end we use *Pythia 2.0* ( Haag & Stamatakis, 2025), a tool for predicting the difficulty of a phylogenetic inference for a given dataset. It predicts a difficulty score between 0 (easy) and 1 (hopeless) using a Gradient-Boosted Tree Regressor ( Haag & Stamatakis, 2025). The predictor provided with the tool is trained on biological data, mainly molecular data. The training data also contains a small proportion of biological morphological data, whose character matrices exhibit similarities to those representing cognate data. To measure the performance of the prediction, ( Haag & Stamatakis, 2025) divides the available datasets into 10 subsets and performs 10 training rounds on 9 of these subsets per round. Thus, during each round, a different subset is omitted from training and subsequently used to evaluate the predictor. Thereby the authors obtain a mean average error (MAE) of 0.08 ( Haag & Stamatakis, 2025). To assess the performance of this predictor on cognate data, we predict the difficulty for the datatsets in Lexibench. Further, we calculate the ground truth difficulty scores as introduced by ( Haag *et al.*, 2022) and obtain an MAE of 0.17. This is substantially higher than the MAE reported by ( Haag & Stamatakis, 2025) on molecular datasets. This shows that we cannot expect the same prediction quality when applying a tool that has mainly been trained on molecular data to language data, presumably because molecular data and cognate data exhibit different properties and behavior ( Häuser *et al.*, 2024b).

In the following we further investigate whether it is possible to train a phylogenetic difficulty predictor from scratch by only using cognate data. Due to the small number of available data sets, we try to use as many of them as possible for training and hence conduct *leave-one-out (LOO) cross-validation* ( Wong, 2015). For each Lexibench dataset under study, we train the predictor separately with all datasets except this one dataset itself. Then, we use the predictor to obtain the difficulty for the very dataset that was left out during training and compare the predicted difficulty to its ground truth difficulty. The experiments yield an MAE of 0.16, which is two times worse than the MAE of 0.08 reported by ( Haag & Stamatakis, 2025) for molecular data. In addition, the improvement of the MAE from 0.17 for the biological data predictor to 0.16 for the dedicated linguistic data predictor is disappointingly small.

The prediction of the difficulty score is based on 10 features ( Haag *et al.*, 2022). Due to the curse of dimensionality, the available cognate datasets may however not suffice to train a reasonable predictor (( James *et al.*, 2023), p. 115). Furthermore, when a predictor is trained on such a small number of datasets, it is possible that the model learns the noise in the data rather than the underlying pattern (( James *et al.*, 2023), p. 152). The reported error is therefore likely to be subject to overfitting.

Hence, tools that have been trained on molecular data can therefore neither be directly applied to cognate data without restrictions nor does the amount of currently available cognate data suffice to re-train these tools. The code for these experiments is available online ( Häuser, 2025c [Code]).

## 5 Conclusion and discussion

In this work we investigated how and if recent advances in molecular phylogenetics can be applied to cognate data from historical linguistics. Namely, we focused on developing and assessing more sophisticated models of language evolution and investigate if machine learning approaches that work well on molecular data can also be applied to language data. In Section 2, we initially provided an overview of the currently available cognate data. We put the amount of data in relation to the available amount of molecular data which is larger by several orders of magnitude. Then, we introduced a novel representation of cognate data and devised two evolutionary models that are tailored to this representation (see Section 3). However, these models are more parameter-rich and, as we quantitatively show, suffer from overparametrization when applied to the currently available relatively small cognate datasets. In addition, by example of the Pythia machine learning tool that is highly accurate on molecular data, we have shown that the available amount of cognate data is also insufficient for training and applying machine learning approaches from the area of molecular phylogenetics (see Section 4). As long as larger datasets are not available, we therefore advocate for using the standard binary representation in conjunction with the corresponding simple evolutionary models. Any tool or approach that relies on machine learning must also be treated with caution when applied to cognate data. This is because the training and, as a consequence, performance of such methods is also severely limited by the small amount of data as well as small number of datasets available. Our work shows that larger datasets and overall more datasets are required before recent advances in molecular phylogenetics can be applied to historical linguistics. To move forward, we therefore recommend focusing on acquiring more data and investigating distinct approaches, sources, and data types as well as combinations thereof.

## Ethics and consent

Ethical approval and consent were not required.

## Data Availability

Unless otherwise stated, our experiments are based on the benchmark data collection *Lexibench* ( Häuser & List, 2025) made available on Zenodo ( https://doi.org/10.5281/zenodo.15804927) ( Häuser *et al.*, 2025a) Experiments related to the models for cognate data: https://github.com/luisevonderwiese/cognate-model https://doi.org/10.5281/zenodo.17748822 ( Häuser, 2025b [Code]) Result tabels for experiments with cognate models: https://doi.org/10.5281/zenodo.17748854 ( Häuser, 2025e [Data]) Experiments related to machine learning using Pythia as an example: https://github.com/luisevonderwiese/pythia_study https://doi.org/10.5281/zenodo.15798101 ( Häuser, 2025c [Code]) Analysis of the available molecular data in *TreeBase* ( Piel *et al.*, 2009): https://github.com/luisevonderwiese/scripts https://doi.org/10.5281/zenodo.15805506 ( Häuser, 2025a [Code]) All data is available under the terms of the Creative Commons Attribution 4.0 International license (CC-BY 4.0).

## Software availability

- PyLexibench for access to Lexibench: https://codeberg.org/lexibank/pylexibench https://doi.org/10.5281/zenodo.15798278 ( Häuser *et al.*, 2025b [Software]) Available under the terms of the Creative Commons Attribution 4.0 International license (CC-BY 4.0).
- RAxML-NG version including the models COG and COGs: https://github.com/luisevonderwiese/raxml-ng-cognate https://doi.org/10.5281/zenodo.15798064 ( Häuser, 2025d [Software]) Available under the terms of the GNU Affero General Public License version 3.
