# Supplementary material for: A systematic exploration of current limitations of cognate-based phylogenetic inference
Source: Open Res Eur. 2026 Jan 28;5:258. Originally published 2025 Aug 27. [Version 3] doi: 10.12688/openreseurope.20351.3 (PMC12859443; doi:10.12688/openreseurope.20351.3)
Supplement: Peer review history

# Reviewer report: Reviewer response for version 3

**DOI:** 10.21956/openreseurope.24814.r68736

**Published:** 2026-01-30

**Authors:** Osama A Salman

**Affiliations:** 1 EET, Budapest University of Technology and Economics (Ringgold ID: 172285), Budapest, Budapest, Hungary

I thank the authors for the additional revision. The wording inconsistency in the description of the cross-validation results has been corrected, and a clear paragraph describing the data inclusion criteria has been added. My previous concerns have been fully addressed, and I now approve the article.

If any, are all the source data and materials underlying the results available?

Yes

Is the work original in terms of material and argument?

Yes

Is the work clearly and cogently presented?

Partly

Is the argument persuasive and supported by evidence?

Partly

Does the research article contribute to the cultural, historical, social understanding of the field?

Yes

Does it sufficiently engage with relevant methodologies and secondary literature on the topic?

Partly

Reviewer Expertise:

My expertise is in computational phylogenetics and data science for scriptinformatics, focusing on model evaluation, cross-validated analysis, and reproducible workflow

I confirm that I have read this submission and believe that I have an appropriate level of expertise to confirm that it is of an acceptable scientific standard.

# Reviewer report: Reviewer response for version 2

**DOI:** 10.21956/openreseurope.23907.r67967

**Published:** 2026-01-21

**Authors:** Osama A Salman

**Affiliations:** 1 EET, Budapest University of Technology and Economics (Ringgold ID: 172285), Budapest, Budapest, Hungary

I thank the authors for the thorough revision. The manuscript has been substantially improved, and my main scientific concerns regarding over-parameterization and the empirical validity of the modeling assumptions are now explicitly acknowledged, tested, and clearly discussed. In its current form, the paper makes a solid and well-supported contribution as a negative-result and methodological caution study, and I consider it **indexable**

 I recommend **approval with minor reservations**, pending two small but important clarifications:

 1- There is an inconsistency in the description of the cross-validation results: Figure 14 refers to errors being “7 orders of magnitude higher,” while the main text correctly describes them as approximately “7 times higher.” This should be corrected for consistency and accuracy.

 2- (Optional) As suggested previously, I still recommend adding a short, explicit paragraph summarizing the dataset and literature search strategy and inclusion criteria (even if a PRISMA figure is provided), to improve transparency and readability.

If any, are all the source data and materials underlying the results available?

Yes

Is the work original in terms of material and argument?

Yes

Is the work clearly and cogently presented?

Partly

Is the argument persuasive and supported by evidence?

Partly

Does the research article contribute to the cultural, historical, social understanding of the field?

Yes

Does it sufficiently engage with relevant methodologies and secondary literature on the topic?

Partly

Reviewer Expertise:

My expertise is in computational phylogenetics and data science for scriptinformatics, focusing on model evaluation, cross-validated analysis, and reproducible workflow

I confirm that I have read this submission and believe that I have an appropriate level of expertise to confirm that it is of an acceptable scientific standard, however I have significant reservations, as outlined above.

# Reviewer report: Reviewer response for version 1

**DOI:** 10.21956/openreseurope.22023.r59919

**Published:** 2025-10-03

**Authors:** Angie S Hinrichs

**Affiliations:** 1 University of California Santa Cruz, Santa Cruz, California, USA

In "A systematic exploration of current limitations of cognate-based phylogenetic inference," Haeuser et al. apply modern phylogenetic methods, specifically the maximum-likelihood phylogenetic tree inference tool RAXML-NG with a variety of evolutionary models, to cognate datasets.  They demonstrate in multiple ways that the size of available cognate datasets is insufficient for accurate phylogenetic inference with methods developed for the large datasets that are available from DNA sequencing, while introducing novel models of cognate evolution that show promise in case the size of cognate datasets should grow much larger in the future.  The data and models are clearly described and are available online from Zenodo and GitHub.

 The novel models of cognate evolution, COG and its simpler variant COG_s, are thoughtfully designed to have a small number of free parameters in order to avoid over-parameterization as much as possible, while allowing some flexibility by having more than zero free parameters as the simplest possible models would have.  They are based on a binary vector encoding of presence/absence of cognate classes in different languages.  Using a binary vector to represent each cognate concept is an advance over using separate binary columns because the binary columns are treated by inference methods as independent although they are not.  However, using a binary vector when many of the possible values are never observed, and mapping the values of that binary vector directly to a symbol space limited to 64 in RAXML-NG, limits the number of cognate classes per concept to 6 (2^^6 = 64).  The distance metric between two binary vector values is the Hamming distance, i.e. the number of 1 bits in the XOR of the two vectors.  I note that if 7-bit vectors are restricted to those with 3 or fewer 1's (i.e. if popcnt(b) <= 3 for all observed b), there are only 64 such values, which could be mapped to the 64 symbols accepted by RAXML-NG; this extension of the scheme could add support for concepts with seven cognate classes, although there may not be enough concepts with 7 classes to make that of any practical use.

 The substitution rate matrix for COG_s is constrained to allow only two free parameters: lambda_+ for a Hamming distance of exactly 1, and lambda_0 for a Hamming distance greater than 1.  lambda_0 is expected to be close to 0 because the authors make the assumption (referred to as S1) that "at most one word can emerge or disappear within a single infinitesimal time step" (section 3.2.3).  There is a counterexample to this assumption in Figure 3: the value of 001 for N and S exists along with 010 for G and D (two bits change) and 110 (three bits change), without any language having the transitional value 011 that would bridge 001 and 010.  Moreover, assumption B3, i.e. "the probability of a state decreases with with increasing [number of 1 bits]", implies that such bridging values would be less likely, seemingly counter to assumption S1.  Nevertheless, assumption S1 is used both to form the expectation that lambda_0 should be close to zero for COG_s (which is not borne out by experiments in section 3.6.1.1) and as the reason to force lambda_0 to zero in COG.  It seems to me that a Hamming distance of 2 would be quite common, because that is the distance between any pair of differing vectors that each have one bit set (for example, between 001 and 010, between 001 and 100, or between 010 and 100).  By assumptions B1 - B3, those vectors with a single bit set should be the most common, so it doesn't seem right to pin the rate of transition between any such vectors to zero.  I wonder how a slightly different version of COG would perform, with lambda_1 for a Hamming distance of 1, lambda_2 for a Hamming distance of 2, and ~zero otherwise -- or perhaps lambda_+ for Hamming distance of either 1 or 2, and lambda_0 otherwise.

 The point that cognate datasets are too small for modern phylogenetic methods is made in many ways: by comparison of dataset size to a single phylogenetic database in section 2, by Akaike Information Criterion score distributions in 3.6.2, by observations about the complete lack of occurrences of many symbols in 3.6.3.2, by decreasing sizes of subsets necessitated by the new models in 3.6.3.3, by ratios of number of concepts to number of languages in 3.6.3.4, by low cross-validation in 3.7, and by calculations of difficulty of phylogenetic inference using the Pythia tool in section 4.  It's hard to imagine how the authors could have been more thorough in finding many paths to the same conclusion that a relatively small dataset does not lend itself to inference methods that were developed for large datasets.

 And now a list of typos or wording suggestions, in the order encountered in the manuscript:

 "Institute of Computer Scienc": Scienc --> Science

 congate --> cognate

 In section 3.2.3, the assumptions render as a bullet list but are then referred to as B1-B3 and S1-S3.  They should be explicitly labeled.

 "an subsequent": an --> a

 "merely not available": I suggest merely --> simply

 "less free parameters": less --> fewer (because parameters are countable; sorry, English is a ridiculous language)

 "P[s1,s2]": P --> R (in sections 3.3.2.1 and 3.3.2.2)

 "underline again the assumption that the models are overparametrized": it seems more like a conclusion than an assumption?

 "We proof this":  proof --> prove

 "7 orders of magnitude": from 0.3 to 0.03, I count only one order of magnitude...?

 "focusing on acquiring more data and investigate distinct": investigate --> investigating

If any, are all the source data and materials underlying the results available?

Yes

Is the work original in terms of material and argument?

Yes

Is the work clearly and cogently presented?

Yes

Is the argument persuasive and supported by evidence?

Yes

Does the research article contribute to the cultural, historical, social understanding of the field?

Yes

Does it sufficiently engage with relevant methodologies and secondary literature on the topic?

Yes

Reviewer Expertise:

Bioinformatics

I confirm that I have read this submission and believe that I have an appropriate level of expertise to confirm that it is of an acceptable scientific standard.

# Response

**Published:** 2025-11-05

**Authors:** Luise Häuser

**Affiliations:** Heidelberg Institute for Theoretical Studies, Heidelberg, Baden-Württemberg, Germany

Thank you very much for your interesting feedback!

 The example data set from Fig. 3 does not contradict S1. We would assume, that some time ago, there was a language with the transitional value 011 - but then one of the congnates disappeared again. 

 Also S1 and B3 do not necessarily contradict each other. The idea is, that transitional states with more cognates can exist, but disappear again more quickly.

 But thank you for your input how to modify COG for future experiments!

 Unfortunately I cannot fix the typo in Computer Scienc(e), it must have happened during post-processing as it is already correct in my manuscript.

# Reviewer report: Reviewer response for version 1

**DOI:** 10.21956/openreseurope.22023.r59356

**Published:** 2025-09-29

**Authors:** Osama A Salman

**Affiliations:** 1 EET, Budapest University of Technology and Economics (Ringgold ID: 172285), Budapest, Budapest, Hungary

Summary

 This article asks why methods that work well in molecular phylogenetics do not transfer cleanly to cognate-based historical linguistics. The authors

1. Show that high-quality cognate datasets are scarce and small
2. Introduce a concept-level bit-vector encoding with two tailored models (COG/COGs) implemented in RAxML-NG
3.  test model adequacy and parameter stability using AIC and cross-validation, and
4. Check whether ML predictors such as Pythia generalize to cognate data. The results point to over-parameterization and unstable estimates even for simpler models at current data scales, with limited gains from ML. The main take-home grow the data before escalating model complexity follows from the evidence presented

Evaluation & required revisions

 • Engagement with methods/literature  [partly]. Add a short “Search strategy & inclusion criteria” paragraph for both datasets and literature (where searched, date accessed, key terms, basic include/exclude rules).  A tiny PRISMA-style flow is sufficient.

 • Clarity  [partly]. Provide a boxed glossary for key terms (ν, κ, κ-subset, ascertainment/Mk, over-parameterization, cross-validated error) and a one-page taxonomy that separates data issues, model issues, and evaluation pitfalls.

 • Evidence [partly]. Include one compact results table that, for representative datasets, lists κ-subset size distributions, concepts:languages ratios, free-parameter counts per model, and AIC/CV summaries, so the stability/over-parameterization claims are visible at a glance.

 • Presentation [minor]. Standardize terminology (“cognate class/set”), mirror persistent identifiers/licences in captions, and enlarge fonts on multi-panel figures.

 Verdict

 Approved with reservations. With the brief search/eligibility note and the compact stability table plus the small presentation fixes the paper will be methodologically transparent, clearly structured, and scientifically sound.

If any, are all the source data and materials underlying the results available?

Yes

Is the work original in terms of material and argument?

Yes

Is the work clearly and cogently presented?

Partly

Is the argument persuasive and supported by evidence?

Partly

Does the research article contribute to the cultural, historical, social understanding of the field?

Yes

Does it sufficiently engage with relevant methodologies and secondary literature on the topic?

Partly

Reviewer Expertise:

My expertise is in computational phylogenetics and data science for scriptinformatics, focusing on model evaluation, cross-validated analysis, and reproducible workflow

I confirm that I have read this submission and believe that I have an appropriate level of expertise to confirm that it is of an acceptable scientific standard, however I have significant reservations, as outlined above.
